# Neurophysics Assessment of the Muscle Bioenergy Generated by Transcranial Magnetic Stimulation

**DOI:** 10.34133/2019/7109535

**Published:** 2019-03-26

**Authors:** Fidias E. Leon-Sarmiento, Alexander Gonzalez-Castaño, Carlos V. Rizzo-Sierra, Juan Aceros, Daniel S. Leon-Ariza, Juan S. Leon-Ariza, Diddier G. Prada, William Bara-Jimenez, Zeng Y. Wang

**Affiliations:** ^1^Smell and Taste Center, Perelman School of Medicine, University of Pennsylvania, Philadelphia, PA, USA; ^2^Human Motor Control Section, NINDS, National Institutes of Health, Bethesda, MD, USA; ^3^Mediciencias Research Group, Louisville, KY, USA; ^4^Universidad Internacional de la Rioja, Spain; ^5^Neurophysics Unit, Corporación Universitaria Minuto de Dios-UNIMINUTO, Colombia; ^6^Charity Association for Person Centered Medicine, Moral Entity, Italy; ^7^School of Engineering, University of North Florida, Jacksonville, FL, USA; ^8^Faculty of Health Sciences, Santander University, UDES, Colombia; ^9^Department of Neurosurgery, Johns Hopkins University, Baltimore, MA, USA; ^10^Harvard T.H. Chan School of Public Health, Harvard University, Boston, MA, USA; ^11^Unidad de Investigación Biomédica en Cáncer, Instituto Nacional de Cáncer, México DF, Mexico; ^12^Neuromuscular Division, Department of Neurology, University of Louisville, Louisville, KY, USA

## Abstract

The content of the rectified motor evoked potential (MEP) induced by transcranial magnetic stimulation (TMS) has ambiguously been assessed without the precision that energy calculation deserves. This fact has misled data interpretation and misguided biomedical interventions. To definitively fill the gap that exits in the neurophysics processing of these signals, we computed, in* Walls (*$\hat{W}$*),* the* bioenergy *within the rectified MEP recorded from the human first digitorum index (FDI) muscle at rest and under isometric contraction. We also gauged the* biowork *exerted by this muscle. Here we show that bioenergy and biowork can accurately and successfully be assessed, validated, and determined in $\hat{W}$ from MEP signals induced by TMS, regardless of knowing the mathematical expression of the function of the signal. Our novel neurophysics approach represents a dramatic paradigm shift in analysis and interpretation of the content of the MEP and will give a true meaning to the content of rectified signals. Importantly, this innovative approach allowed unveiling that women exerted more bioenergy than men at the magnetic stimulations used in this study. Revisitation of conclusions drawn from studies published elsewhere assessing rectified EMG signals that have used ambiguous units is strongly recommended.

## 1. Introduction

Assessment of the area of full-wave rectify neural signals has been diverse, contradictory, and confusing. V*∗*msc, mV/sec, mV*∗*sec, ms*∗*nM, uV, uv^2^, or just arbitrary units have been some of the miscellaneous units used to calculate the rectified motor evoked potential (MEP) [1, 2], which reflects the corticospinal tracts state from brain to muscles following transcranial magnetic stimulation (TMS) [3, 4]. Since full wave rectification transforms raw signals recorded out of the baseline to a single polarity [5] and indicates the energy contained by a signal [1, 6] it is rather surprising that the aforementioned ambiguous approaches have been used for decades in MEP data processing without the precision energy calculation deserve misleading interpretations and clouding data reproducibility.

To definitively fill the gap that exist on the computation of the energy contained within the MEP, mainly in those cases where the mathematical expression of the function needed to calculate the integral of the signal is unknown, a three-step research was performed. First, we assessed the MEP* bioenergy* exerted by FDI muscle by integrating the squared rectified neural responses recorded during a period of time using the mathematical principle known as Simpson's rule [7]. Second, we calculated bioenergy in $\hat{W}alls\;\;(\hat{W})$, which differs from classical concepts of energy canonically measured in Joules [8] (see methods). Third, we extracted the bioenergy found while the FDI muscle was at rest from that exerted while the same muscle did a voluntary contraction to compute mechanical* biowork*.

We hypothesize that the* bioenergy,* calculated in $\hat{W}$, from the rectified MEP will give more meaningful information and will be better aligned with physics principles of energy conservation than the unspecific assessment made until now in ambiguous units. Since the amount of energy needed to move a muscle represents the mechanical work done by such muscle, we also conjectured that* biowork* can be known by extracting the bioenergy found while the muscle was at rest from that exerted while doing a voluntary contraction. Our results demonstrate the feasibility of using a numerical method to compute both the* bioenergy *and the* biowork *in $\hat{W}$ from a hand muscle. Moreover, they may help to reorient signal processing analyses as well as future research of muscle biomechanics.

## 2. Results

### 2.1. Basic TMS Parameters

All participants tolerated the procedures with no side effects. The mean age of the participants was 49.3 ± 6.5 years (range: 43 - 55 years). There were no age differences between genders (p = 0.053, t-value = -2.035, DF = 25). The resting MEP threshold in the whole group of participants was 48.5 ± 7.9. The motor thresholds (MTs) were normally distributed (*ϒ* = 0.02) and did not show gender differences (males, 47.1 ± 8.3 versus females, 49.9 ± 7.3, p = 0.190; t-value = 1.34; DF: 25).

### 2.2. Method Validation

Perfect correlations were found between the rectified MEP values obtained at rest (r: 0.99) and during voluntary FDI muscle contraction (r: 0.99) using ambiguous* unsquared *units measured by Simpson's rule and the ones captured by Signal® software. These facts validated the accuracy of the bioenergy and biowork assessments we made and detailed in the following sections (*Supplementary Figures *[Supplementary-material supplementary-material-1], [Supplementary-material supplementary-material-1], [Supplementary-material supplementary-material-1]*, and *[Supplementary-material supplementary-material-1]).

### 2.3. Bioenergy Assessment

**Figure 1:**
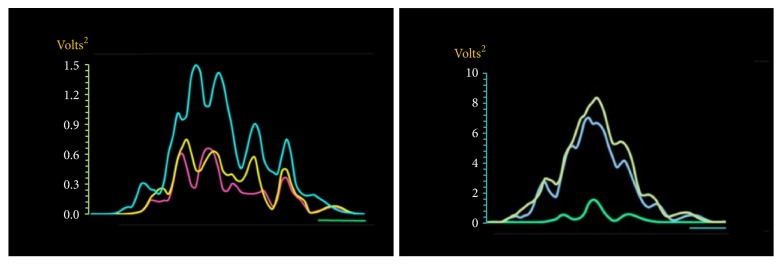
(a)* (left)*. Composite MEPs (cMEP) that delineates the bioenergy obtained from the FDI muscle are rest. The waveforms that encircled the bioenergy showed a remarkable consistency among the discrete cMEPs elicited at MT (purple), MT+10 (yellow), MT+20 (aquamarine). Green horizontal bar: 100 milliseconds. (b)* (right)*. Composite MEPs (cMEP) that delineates the bioenergy obtained from the activated FDI muscle. The waveforms that encircled the bioenergy computed during muscle contraction showed a remarkable consistency among the discrete cMEPs elicited at MT (Green), MT+10 (light blue), and MT+20 (light yellow). It should be noted that the mentioned consistency showed a trend different to the one extracted from the bioenergy computed while the FDI muscle was studied at rest. Blue horizontal line: 100 milliseconds.

**Figure 2:**
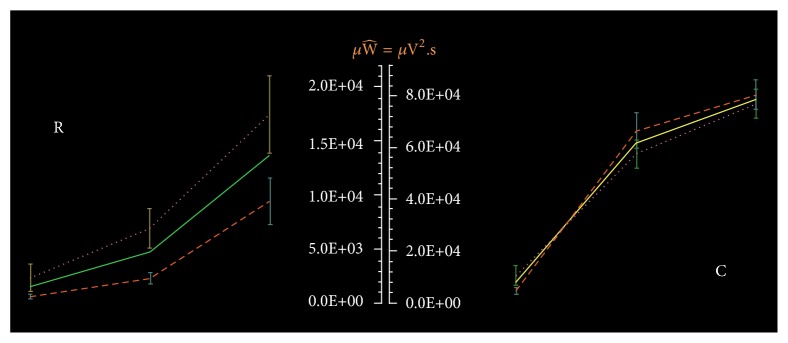
Average of bioenergy (mean ± SE) exerted by applying TMS over the dominant brain motor cortex while the participants had the FDI muscle at rest (R) and during voluntary contraction (C). The bioenergy is greater in females than males at all stimulation intensities at rest (R), and during muscle contraction (C) at MT (see text and Supplementary material [Supplementary-material supplementary-material-1]). X-axis: left, middle, and right error bars represent the TMS applied at MT, MT+10, and MT+20. Males: broken line; females: dotted line; all participants: continues line. The bioenergy is expressed in microWalls.

An ANOVA with group as the main factor found that stimulus intensity modulated bioenergy at rest (p < 0.001; F = 22.81; DF = 2) and during muscle contraction (p < 0.001, F=120, DF=2). The bioenergy found at muscle rest increased significantly during muscle activation (p < 0.001, F: 47.98, DF: 5) (Figures 1(a) and 1(b)). Despite the similar average MT between genders, bioenergy in females was much greater at all stimulus intensities than in men (DF: 3; F: 167.5, P < 0.001) (Figure 2) while the dominant FDI muscle was relaxed. The overall analysis that included male and female data found no significant age effects on the bioenergy measured with the FDI muscle relaxed (p = 0.081, F: 1.39, DF: 66).

ANOVA also showed bioenergy differences among all intensities tested while the participants did an isometric voluntary contraction of FDI muscle (p < 0.001, F = 176.3, and DF = 3) (Figure 2). Significant bioenergy differences were also evident between genders during muscle activation (F: 124.5; p < 0.01, DF = 3); noteworthy, bioenergy was particularly greater in females at MT during muscle contraction (p = 0.005, t-value = 3.141, and DF = 23) (Figure 2, Supplementary [Supplementary-material supplementary-material-1]). Age did not influence bioenergy measured during muscle contraction (p = 0.063, F = 3.63, and DF: 66). Bonferroni corrected post hoc pairwise comparisons confirmed that rectified MEP areas from females contained more bioenergy at rest (p = 0.081, F = 1.39, and DF = 66) and during muscle activation (p = 0.079, F = 1.4, and DF = 66).

MEP bioenergy of the FDI muscle measured in $\hat{W}$ significantly differed from the rectified MEP area measured by ambiguous* unsquared* volts times seconds. These differences were statistically evident when the FDI muscle was studied at rest (p = 0.04, DF: 3, and F: 6.96) and during voluntary contraction (p = 0.04, F: 6.96, and DF: 3).

### 2.4. Biowork Assessment

**Figure 3:**
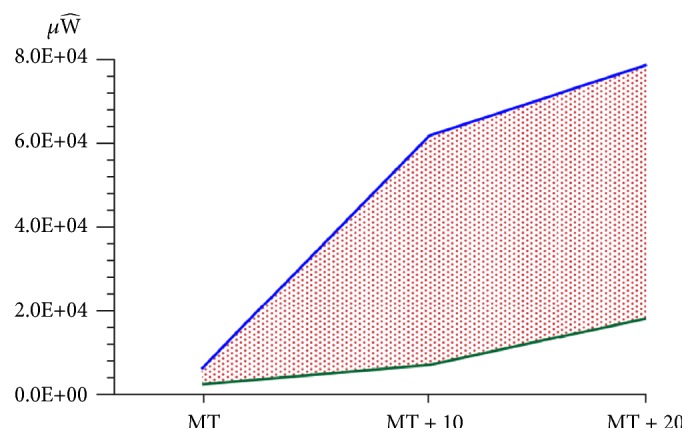
Depiction of the FDI muscle biowork (dots) obtained by applying TMS at motor threshold (MT), ten percent above motor threshold (MT+10), and twenty percent above the motor threshold (MT+20) to the contralateral brain motor cortex of the participants. The stimulus intensities were plotted against the bioenergy obtained with the FDI muscle at rest (green line) and under voluntary muscle contraction (blue line). The biowork (dots) is expressed in microWalls (*μ*$\hat{W}$).

Biowork (Figures 2 and 3*, Supplementary Table *[Supplementary-material supplementary-material-1]) correlated with FDI muscle activation (r = 0.988) and with the muscle relaxed (r = 0.618). ANOVA dissected biowork differences by stimulus intensity (p < 0.001, F = 87.5, and DF = 2) and gender (p < 0.005, F = 148.7, and DF: 3). Biowork was greater in women than in men at MT (p= 0.013, t: 2.68, and DF: 23). Age of participants did not covary (p = 0.688, F = 0.8, and DF = 36). MT did not correlate with biowork (p = 0.58, r = 0.08).

## 3. Discussion

We demonstrate for the first time that bioenergy and biowork are generated by and can be measured from the FDI muscle using confidently $\hat{W}$ regardless of waveform complexity or knowledge of the mathematical function that originates the MEP recordings. This novel approach of MEP energy would overcome issues found in classical calculation of electromyographic parameters such as latencies, duration and dispersion, among others, originated by misplacing cursors over the recordings [9]. Indeed, significant differences were found between bioenergy and biowork parameters assessed in $\hat{W}$ and measures using ambiguous units. Of remark, FDI female muscle generated more $\hat{W}$ than males.

Although this study was not planned to disentangle the physiology of MEP (*Supplementary Discussion*), gender differences of bioenergy are not a matter of brain or body size, corticospinal integrity and connectivity, aging, or hormone females [9–11]. Our results indicate that large muscles such as those found in some men not necessarily contain more bioenergy than short muscles classically found in some women [12]. These findings may reflect the daily preferential use and exercise of the dominant FDI muscle made by women during thousands of years; this exercise would induce more effective behavioral adaptations at lower firing rates than those in males [10–12]. Whatever the mechanisms could be, this study clearly demonstrated that women are stronger and, sometimes, more efficient than men, at least in some neural connections.

In practice, the assessment of muscle bioenergy and mechanical biowork under different lengthening and shortening conditions, in a range of safe TMS intensities, would be useful to, for example, refine countermeasures aiming to improve the performance of healthy people [13]. Likewise, the novel bioenergy assessment dissected here will serve to tailor rehabilitation measures aiming to modulate the aberrant plasticity originated by disorders clinically characterized by lack of energy such as movement disorders, stroke, and spinal cord injury, among others [14, 15].

Our method represents a paradigm shift in assessment, analysis, and interpretation of MEP bioenergy giving altogether full mathematical meaning to the content of rectified signals [2, 6]. Counting as accurate as possible the bioenergy of the MEP will reduce uncertainty, improve outcomes, enhance internal validity, boost external validity, and ensure reproducibility of studies aiming to better understand the motor system function of living organisms. $\hat{W}$ calculation can be useful to accurately determine parameters such as oxygen consumption and ATP, among other variables used to define energy muscle expenditures. Likewise, $\hat{W}$ can be used to better design experiments and tailor countermeasures aiming to develop more focused strategies planned to ameliorate muscle fatigue. Revisitation of conclusions drawn from studies publishing rectified signals data using ambiguous units is strongly recommended.

## 4. Methods

### 4.1. Neurophysics Units for Bioenergy Computation

Since the main aim of this work was to numerically assess the energy within a rectified MEP, the fundamentals of physics that allow computing the bioenergy contained within that MEP should be defined [16]. In brief, the magnitude of the MEP is measured in Volts, which are defined as the energy consumed per electric charge. Energy is, on the one hand, measured in Joules and defined as the capacity for doing work, which relates to the force expended to move particles from one place to another. Charge is, on the other hand, a physical property of some particles that is expressed in Coulomb. Should the energy be consumed during an interval of time it will make power, which is function of both voltage and current. Current is, in turn, the relationship of the movement of particles to voltage, whereas the dimension that expresses the antagonism expended to avoid the movement of the same particles is known as resistance. In an alternate circuit such as the one used by TMS stimulators, resistance is interchangeable with impedance both of which are, in practice, expressed in Ohms. Since impedance is too high in biological tissues, the energy induced by the TMS magnetic field was found undistorted by some authors [17]. Having said this, the aforementioned dimensions will mathematically be expressed, empirically verified, and algebraically solved following physics principles as follows:

### 4.2. Elucidation of Physics Principles of Bioenergy

Energy (*E*) is defined as Power (*P*) multiplied by time (*t*):

(1) $$\begin{array}{c} E=P∙t \end{array}$$

*E* units are expressed in* Joules* (*J*),* P* units are expressed in* Watts* (*W*), and **t** units are expressed in* seconds* (*s*).

By reordering terms, we have

(2) $$\begin{array}{c} P=\frac{E}{t} \end{array}$$

*P *equals voltage (*V*) times current (*I*), or

(3) $$\begin{array}{c} P=V∙I \end{array}$$

*V* units are expressed* Volts* (*V*);* I* units are expressed* Amperes* (*A*)

Ohm's law establishes that* V* is proportional to* I*. This proportion is assumed as constant, and it is named as resistance (*R*):

(4) $$\begin{array}{c} V=I∙R \end{array}$$

Resistance units are expressed* Ohms* (Ω).

From Ohm's Law, current* I* can be written as

(5) $$\begin{array}{c} I=\frac{V}{R} \end{array}$$

By replacing (5) in (3), we have (6):

(6) $$\begin{array}{c} P=V\left(\;\frac{V}{R}\;\right)=\frac{V^{2}}{R} \end{array}$$

Replacing (2) in (6) we have (7):

(7) $$\begin{array}{c} \frac{E}{t}=\frac{V^{2}}{R} \end{array}$$

By reordering terms, we will have

(8) $$\begin{array}{c} E∙R=V^{2}∙t \end{array}$$

Units in (8) are Joules multiplied by Ohms equals* squared Volts multiplied by seconds* or *J*∙Ω = *V*^2^∙*s*

Then,* bioenergy* can be calculated using the integral in

(9) $$\begin{array}{c} \overset{t_{f}}{\underset{t_{o}}{\int }}{\left[\;f\left(\;t\;\right)\;\right]}^{2}dt \end{array}$$

where **f**(**t**) is the function of the recorded MEP. **f**(**t**) units are in volts (**V**), classically measured in the Y-axis. Therefore, [**f**(**t**)]^2^ units are **V**^2^, or squared volts, and multiples. The units of the differential **dt** are seconds (**s**), classically measured in the X-axis. Hence, the integral argument [**f**(**t**)]^2^**dt** units are* squared volts multiplied by seconds*.

These mathematical principles demonstrate that the* bioenergy* computed from neural responses stands apart from the classical concept of energy (**E**) (see (1)), since **E** units are canonically expressed in Joules. Having said this,* bioenergy* will be expressed as $\hat{W}$ that equals* squared volts multiplied by seconds*.

### 4.3. Participants

The sample size was calculated using OpenEpi software. The power was predetermined in 80% and *α* = 0.05. 48 participants were recruited from the Maryland, Virginia, and metropolitan Washington, DC area, USA. Potential participants underwent a thorough clinical neurological exam to rule out possible clinical or subclinical central (e.g., depression, cognitive disorder) or peripheral (e.g., neuropathy, myopathy) nerve system anomalies. Only individuals without neurological deficits were included in this study. Individuals had to be free of medications, ongoing alcohol, and drug abuse. People participating in a research study that might interfere with the results of the proposed study were also excluded.

Exercise and beverages including alcohol and caffeine beverages were avoided for 12 hours before this study. The participants had to be free of recreational drugs by medical history and should not be current smokers. The studies started two to three hours after breakfast. Individuals were asked to remain seated during the experiments and try their best to avoid any mental effort. Anovulatory females who naturally stopped having menstrual periods five years before the testing and had not hot flashes and sleep problems after stopping the menstrual period, were included in the study. This approach avoided the influence of hormones in TMS measures [18]. Skin temperature of participants was kept between 32°C and 36°C.

### 4.4. Magnetic Stimulation

Following validated international guidelines issued by the International Federation of Clinical Neurophysiology for TMS research [19], the magnetic pulses delivered via a Bistim module were applied over the brain motor cortex through a circular 9 cm diameter coil, which is less susceptible to minor changes in the coil position than figure-of-eight coils [19]. The TMS coil was held tangentially near the vertex in the optimal position and orientation for stimulating M1 of the FDI muscle of the dominant hand. The current flowed counterclockwise when viewed from above.

### 4.5. Equipment Set-Up

The EMG activity was recorded in a standard office computer and stored for offline analysis. The recordings were amplified, analog filtered (100/1000 Hz), and digitized at a sampling frequency of 2 kHz. Digitization was done with a Micro 1401 interface. MEPs obtained at rest and under voluntary contraction were analyzed under Signal® software (Cambridge Electronic Design; ced.co.uk).

### 4.6. Muscle to Be Studied

The dominant FDI muscle was chosen to record the MEP responses. It was determined because the discharge of dominant motor unit variability of the FDI muscle is much less than that recorded from the nondominant muscle [20]. Further, FDI muscle is composed of a mixed fiber-type muscle whose majority of fibers are type I [21], which are the main type of fibers used in most daily activities [22]. The fiber configuration of this muscle lever system fits in the bore of a magnet for simultaneous mechanical and energetic measurements during muscle contraction [9]. These aspects make this muscle unique for studying biomechanics [23].

### 4.7. MEP Recordings

Skin preparation technique was made following standard methods and cleaned with alcohol. Skin impedance was set up at 5 kΩ, was checked before MEP recordings, and was kept constant during the experiments. The MEPs were recorded from the dominant FDI muscle both during relaxed and under voluntary contraction. MEP threshold was defined as the lowest stimulation intensity producing MEPs of at least 50 *μ*V, in 5 out of 10 consecutive stimulations. Then, ten single stimuli were applied over the contralateral hot spot of the brain motor cortex that controlled the dominant FDI muscle. The stimuli were delivered at motor threshold (MT), at 10% (MT+10), and at 20% (MT+20) above the MT intensity. These intensities guaranteed activation of both spinal motor neurons and the cerebral cortex, avoid MEP saturation, induce less variability of the muscle response, and avoid intensity-associated potential hazards [24].

One week later, participants returned to do the TMS study while they were doing a muscle effort. TMS intensities and repetitions were the same as those used while the subjects were relaxed. Individuals were instructed to exert a steady isometric contraction of the FDI muscle against a strain gauge to 25% of maximum strength to undergo activation and generate force [25]. This muscle strength was chosen since it is within the range where the great majority of human functional and expressive tasks are performed [8]. The participants were instructed to begin muscle contraction about three seconds before TMS and to maintain the contraction until they were told to relax immediately after the MEP was visualized on the monitor screen. This procedure minimized fatigue and maintained muscle relaxation, ensured by monitoring the activity of the right orbicularis oculi muscles. Participants were asked to keep the eyes open during the experiments to prevent unwanted downregulation of visuomotor inhibitory mechanisms [25].

### 4.8. Region of Interest (ROI) Treatment

The MEP signals were full wave rectified over the horizontal or X-axis. Then, the duration of the ROI, calculated in seconds, was delimited by two vertical cursors placed over the X-axis. One of these two vertical cursors was placed at the time when the MEP activity deflection within the ROI started. This time was named as t_0_. The second vertical cursor was placed over the time when the MEP activity ended, and a flat baseline starts. This time was named as **t**_**f**_. Assume now a virtual third vertical cursor moving from **t**_0_ to **t**_**f**_. For every time **t** on the ROI this virtual cursor intersected the MEP recorded. Such intersection point represented the MEP voltage that was measured in volts or multiples, over the Y-axis. As a result, there was a function **V**(**t**) that gave the voltage magnitude of the MEP for each time **t**. Thus, pairs of data (**t**, **V**(**t**)) were obtained every moment **t** varied from **t**_0_ to **t**_**f**_ within the ROI. These data pairs were exported as text files to a Microsoft excel spreadsheet.

### 4.9. Method Validation

Since a new way of assessing the MEP content is introduced in our research, we considered that it would be appropriate to validate the method used to compute bioenergy of the MEP with the most current alternative, or existing gold standard test. Thus, by using Simpson's rule, we calculated the area from the same rectified MEPs used to compute the bioenergy using, at this time, the ambiguous units reported by the majority of studies, indeed unsquared volts times seconds, and multiples [2, 26]. The following steps were ensued.

First, the ROI of the MEP responses plotted in Signal® were selected as mentioned in the ROI* treatment section. *This region was delimited by the interval [**t**_0_, **t**_**f**_]. After this, the values obtained in ambiguous units of volts times seconds given automatically by the Signal® software were saved in an excel spreadsheet (*Supplementary Figure *[Supplementary-material supplementary-material-1]).

Then, the numerical data of the ROI from each file delineated by the Signal® software were exported as a text file to an excel spreadsheet.

Second, a symmetrical partition of the interval [**t**_0_, **t**_**f**_] was made. As a result, the following dataset was obtained:

(10) $$\begin{array}{c} \left\{\;t_{0},t_{1},t_{2},\cdots ,t_{n-2},t_{n-1},t_{n}\;\right\} \end{array}$$

where **t**_**n**_ = **t**_**f**_. Thus, the number of data within the selected interval was equal to **n** + 1. Caution was taken to have an even number of **n** to properly apply Simpson's rule. The subscript **n** of the dataset represents the number of symmetric subintervals [**t**_**i**−1_, **t**_**i**_] whose width, or ∆**t**, is equal to **t**_**i**_ − **t**_**i**−1_. Subscript **i** is any value in the sequence 1,2,3,···,* n. *Δ**t** is also obtained using the formula Δ**t** = (**t**_**f**_ − **t**_0_)/**n**.

Third, area of the rectified MEP was calculated using the integral

(11) $$\begin{array}{c} \overset{t_{f}}{\underset{t_{o}}{\int }}f\left(\;t\;\right)dt \end{array}$$

where **f**(**t**) is the unknown mathematical expression of the function of the MEP signal. **t** is the time delimited by ROI. The integral can be solved using the rule of Simpson as follows:

(12) ∆t3ft0+ft1+2ft2+4ft3+⋯+2ftn−2+4ftn−1+ftn

where every **f**(**t**_**i**_), for **i** = 0 to **n**, is the voltage that correspond to every time at **t**_**i**_. Using Signal®, we found that, at a frequency of 2 kHz, Δ**t** of 0.0005 seconds selected in the interval [**t**_0_, **t**_**f**_] was enough to guarantee convergence of the numerical method to the real value of integral.

When the number of data obtained from the ROI selected was even, the next numerical datum after **t**_**f**_ was included at the end of data set, extending the time interval in 0.0005 seconds.

### 4.10. Bioenergy Assessment

Classical algebraic principles establish that to solve any integral the mathematical expression of the function must be known [27]. In our case, the mathematical expression of the function that allowed recording the MEPs was unknown. Therefore, the following steps were ensued to assess bioenergy using Simpson's rule.

First, the ROI of the MEP responses plotted in Signal® were selected as mentioned in the section of ROI treatment. This region was delimited by the interval [**t**_0_, **t**_**f**_]. Then, the numerical data from each file were exported as a text file to an excel spreadsheet.

Second, a symmetrical partition of the interval [**t**_0_, **t**_**f**_] was made. As a result, the following dataset was obtained:

(13) $$\begin{array}{c} \left\{\;t_{0},t_{1},t_{2},\cdots ,t_{n-2},t_{n-1},t_{n}\;\right\} \end{array}$$

where **t**_**n**_ = **t**_**f**_. Thus, the number of data within the selected interval was equal to **n** + 1. Caution was taken to have an even number of **n** to properly apply Simpson's rule. The subscript **n** of the dataset represents the number of symmetric subintervals [**t**_**i**−1_, **t**_**i**_] whose width, or ∆**t**, is equal to **t**_**i**_ − **t**_**i**−1_. Subscript  **i**  is any value in the sequence 1,2, 3, ···, *n*. Δ**t** is also obtained using the formula Δ**t** = (**t**_**f**_ − **t**_0_)/**n**.

Third, bioenergy was calculated using the integral in (9):

(14) $$\begin{array}{c} \overset{t_{f}}{\underset{t_{o}}{\int }}{\left[\;f\left(\;t\;\right)\;\right]}^{2}dt \end{array}$$

where **f**(**t**) is the unknown mathematical expression of the function of the MEP signal. **t** is the time delimited by ROI. The integral can be solved using the rule of Simpson as follows:

(15) ∆t3ft02+ft12+2ft22+4ft32+⋯+2ftn−22+4ftn−12+ftn2

where every **f**(**t**_**i**_), for **i** = 0 to **n**, is the voltage that corresponds to every time at **t**_**i**_. Each value for **f**(**t**_**i**_) must be squared before calculation. Using Signal®, we found that, at a frequency of 2 kHz, Δ**t** of 0.0005 seconds selected in the interval [**t**_0_, **t**_**f**_] was enough to guarantee convergence of the numerical method to the real value of integral (see results).

When the number of data obtained from the ROI selected was even, the next numerical datum after **t**_**f**_ was included at the end of data set, extending the time interval in 0.0005 seconds.

### 4.11. Bio-Work Assessment

**Figure 4:**
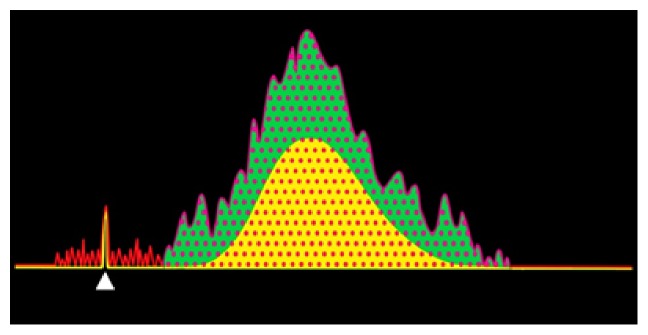
Depiction of biowork (green area) that results by subtracting the bioenergy exerted by a muscle at rest (yellow) from the bioenergy (dotted area) obtained from the same muscle during voluntary contraction (red). White arrowhead: TMS artifact.

Work in physics is defined as how much energy is being used to move something [28]. Accordingly, we aimed to know the biowork exerted by the FDI muscle by subtracting the bioenergy obtained during muscle at rest from the one obtained while doing a muscle contraction (Figure 4). Bioenergy of the MEP recorded during a muscle contraction was measured in a similar way as the bioenergy assessment done on the MEPs recorded at rest with a variation. **t**_**f**_ was at this occasion placed at the time when the muscle activation ended followed by a flat baseline [2, 29].

### 4.12. Statistical Analyses

The order of the TMS intensities was carefully randomized within and across subjects to avoid order effects and hysteresis in the input-output motoneuron recruitment. Kurtosis (*ϒ)*, a test based on numerical methods validated to study data distribution in small and large samples, was used to assess data normality [30]. Sphericity was run and confirmed using the Mauchly test. Two-sample comparisons were performed with t-tests. An analysis of variance (ANOVA) was run to compare the bioenergy measured in $\hat{W}$*alls* with the ambiguous unsquared rectified MEP area measured by Simpson's rule as well as Signal®. Bioenergy data were also subjected to ANOVA repeated measures to analyze bioenergy by two states (rest versus activity) and three types of stimulation (intensity). The data from rectified MEP area obtained at different stimulus intensities were also subjected to analysis of covariance (age = covariate) with the between-subject factor of gender (males versus females). Because MEP threshold could have driven differences in the biowork, we looked for correlations coefficients (r) between it and the MT. For this purpose, we collapsed the data across all stimulus intensities and took the mean value of the MEP data. Post hoc analyses were carried out using Bonferroni's comparison. Pearson correlation coefficients (r) were also run for MEP data obtained in ambiguous units of volts times seconds by applying the Simpson's rule and Signal®. P value was set at < 0.05. SPSS 24® for Windows was used to do the statistical analyses.

## Data Availability

All data needed to support the conclusions of this study are available within paper and the Supplementary Materials. Additional data related to this paper may be requested from the corresponding author upon reasonable request.

## Supplementary Materials

Supplementary Materials
**Supplementary Figure** **1**. Graphical consistency of the methods used to calculate the area of the rectified MEP in ambiguous units or unsquared millivolts times seconds. Horizontal bars to the left: data obtained with the FDI muscle at rest (R). Horizontal bars to the right: data obtained with the FDI muscle contracted (C). Brown bars: MT, purple bars: MT+10, green bars: MT+20. Dashed bars: values obtained by Simpson's rule. Filled bars: values given by Signal® software. The coefficient correlation between the two methods was 0,99.** Supplementary Figure****2**. Correlation coefficients (r) calculated between the assessments made by Simpson's rule (X-axis) and Signal® (Y-axis) using the ambiguous units of millivolts times milliseconds. r: resting muscle; a: active muscle.** Supplementary Figure****3**. Graphical depiction of the validation method made to the area of the rectified MEP in ambiguous units, or unsquared volts times seconds, obtained from a healthy human with the FDI muscle at rest. White vertical lines: region of interest. SS: values obtained automatically from Signal software®, SR: values obtained by applying the Simpson's rule. The correlation coefficient (*r*) obtained between these two methods was 0,99 (see text). Green arrowhead: magnetic pulse.** Supplementary Figure****4**. Graphical depiction of the validation made to the area of the rectified MEP in ambiguous units, or unsquared volts times seconds, obtained a healthy human with the FDI muscle contracted. White vertical lines: region of interest. SS: values obtained automatically from Signal software®, SR: values obtained by applying Simpson's rule. The correlation coefficient (r) obtained between these two methods was 0,99 (see text). Green arrowhead: magnetic pulse.** Supplementary Figure****5**. Example of the numerical method followed to validate the computation of the area of the rectified neural signal using ambiguous units, or unsquared volts times seconds. Numbers highlighted in yellow color (left column) correspond to the region of interest selected to calculate the area of the rectified MEP. Numbers highlighted in green, blue and pink correspond to the values obtained to calculate the area by using Simpson's rule, which is depicted in dark blue color. A screenshot of the Signal® software is also inserted that included the most salient information automatically given by the software used to know the area of the rectified MEP. Time: green line, voltage: blue line, area measured in ambiguous units, or volts times seconds: red line. Note that the values obtained in ambiguous units, or volts times seconds, of the area of the rectified MEP obtained by the Signal® software (numbers in purple color) and those obtained by Simpson's rule (numbers in red color) were similar and strongly correlated (r:0,99, see text). In brown color: t: time in seconds, V: voltage in volts, ||: absolute value.** Supplementary Table**: data obtained in absolute values (AV), and in decadic logarithm (Log 10) transformed.
