# Supplementary material for: Neurophysics Assessment of the Muscle Bioenergy Generated by Transcranial Magnetic Stimulation
Source: Research (Wash D C). 2019 Mar 26;2019:7109535. doi: 10.34133/2019/7109535 (PMC6750091; doi:10.34133/2019/7109535)
Supplement: Supplementary Materials — Supplementary Figure 1. Graphical consistency of the methods used to calculate the area of the rectified MEP in ambiguous units or unsquared millivolts times seconds. Horizontal bars to the left: data obtained with the FDI muscle at rest (R). Horizontal bars to the right: data obtained with the FDI muscle contracted (C). Brown bars: MT, purple bars: MT+10, green bars: MT+20. Dashed bars: values obtained by Simpson's rule. Filled bars: values given by Signal® software. The coefficient correlation between the two methods was 0,99. Supplementary Figure2. Correlation coefficients (r) calculated between the assessments made by Simpson's rule (X-axis) and Signal® (Y-axis) using the ambiguous units of millivolts times milliseconds. r: resting muscle; a: active muscle. Supplementary Figure3. Graphical depiction of the validation method made to the area of the rectified MEP in ambiguous units, or unsquared volts times seconds, obtained from a healthy human with the FDI muscle at rest. White vertical lines: region of interest. SS: values obtained automatically from Signal software®, SR: values obtained by applying the Simpson's rule. The correlation coefficient (r) obtained between these two methods was 0,99 (see text). Green arrowhead: magnetic pulse. Supplementary Figure4. Graphical depiction of the validation made to the area of the rectified MEP in ambiguous units, or unsquared volts times seconds, obtained a healthy human with the FDI muscle contracted. White vertical lines: region of interest. SS: values obtained automatically from Signal software®, SR: values obtained by applying Simpson's rule. The correlation coefficient (r) obtained between these two methods was 0,99 (see text). Green arrowhead: magnetic pulse. Supplementary Figure5. Example of the numerical method followed to validate the computation of the area of the rectified neural signal using ambiguous units, or unsquared volts times seconds. Numbers highlighted in yellow color (left column) correspond to the [file 7109535.f1.pdf]

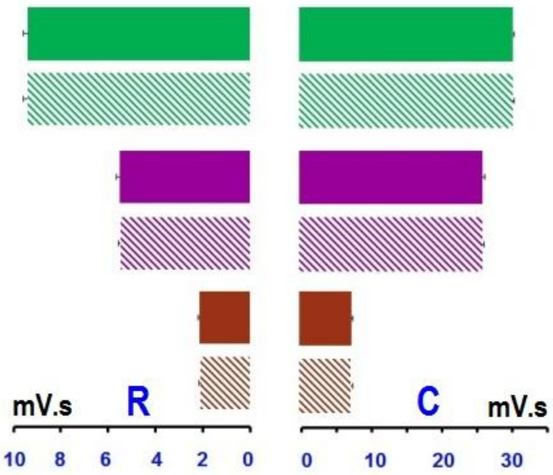

Supplementary Fig. 1. Graphical consistency of the methods used to calculate the area of the rectified MEP in ambiguous units, or unsquared millivolts times seconds. Horizontal bars to the left: data obtained with the FDI muscle at rest (R). Horizontal bars to the right: data obtained with the FDI muscle contracted (C). Brown bars: MT, purple bars: MT+10, green bars: MT+20. Dashed bars: values obtained by the Simpson´s rule. Filled bars: values given by Signal® Software. The coefficient correlation between the two methods was 0,99.

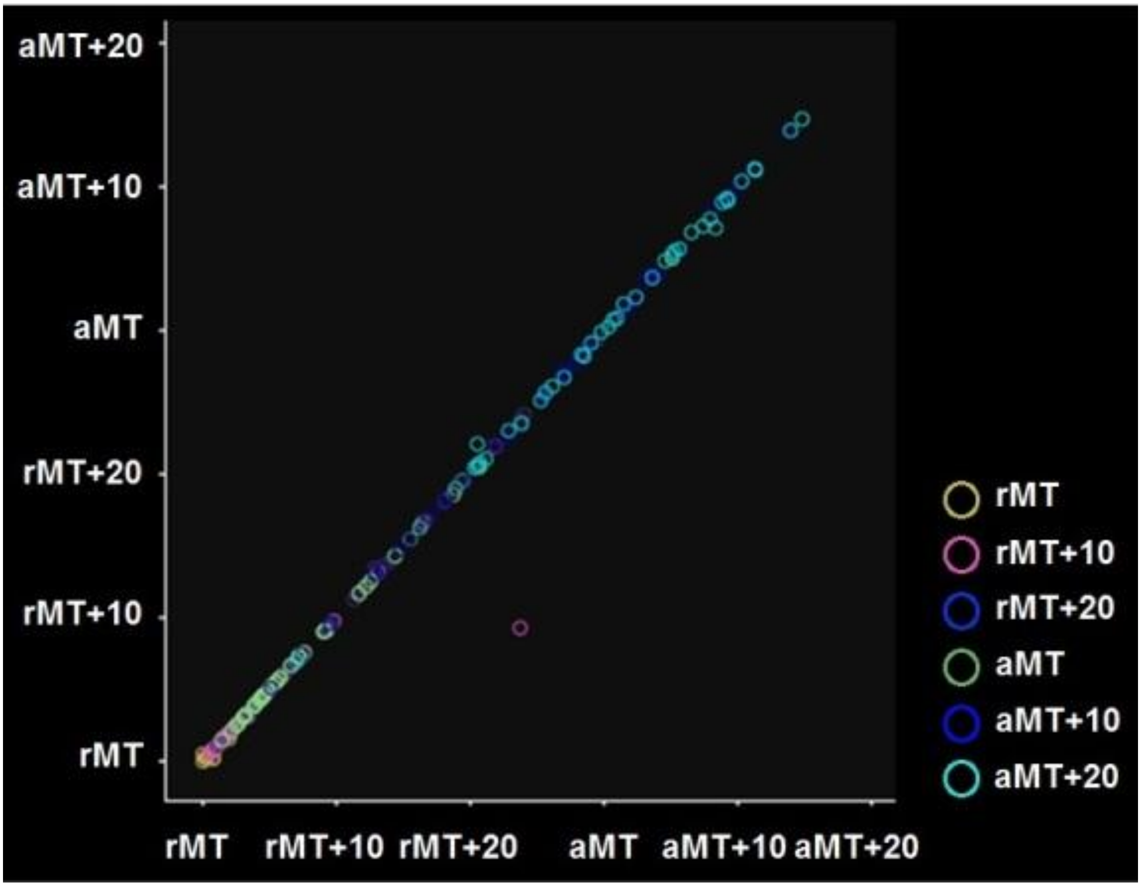

Supplementary Fig. 2. Correlation coefficients (*r*) calculated between the assessments made by the Simpson's rule (X-axis), and Signal® (Y-axis) using the ambiguous units of millivolts times milliseconds. r: resting muscle; a: active muscle.

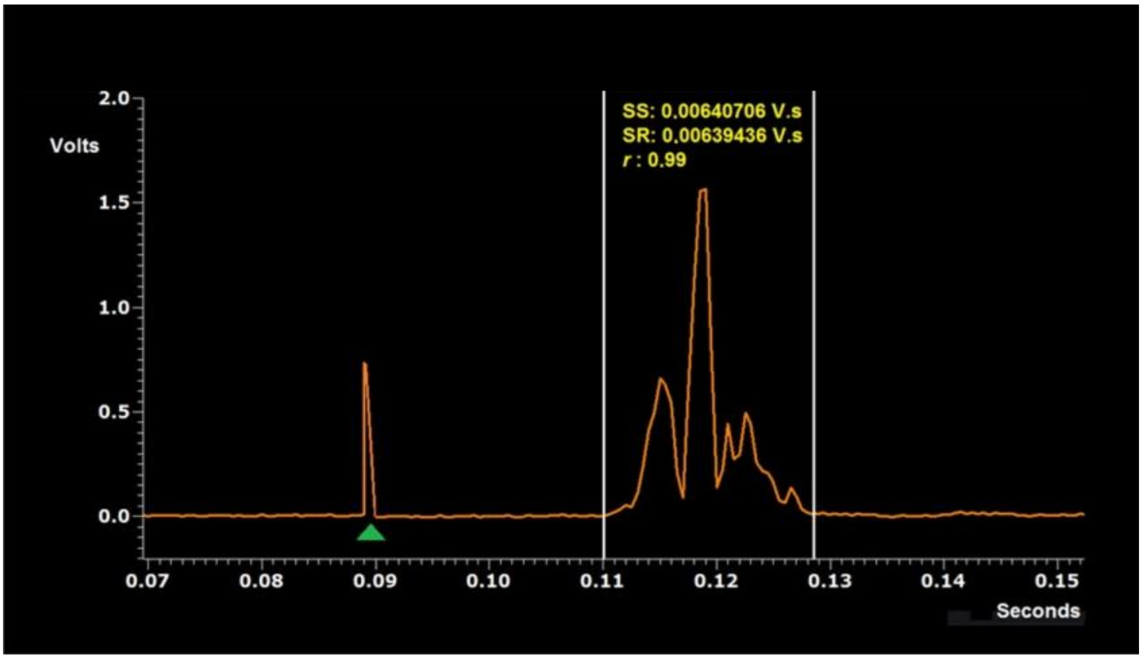

Supplementary Fig. 3. Graphical depiction of the validation method made to the area of the rectified MEP in ambiguous units, or unsquared Volts times seconds, obtained from a healthy human with the FDI muscle at rest. White vertical lines: region of interest. SS: values obtained automatically from Signal software®, SR: values obtained by applying the Simpson's rule. The correlation coefficient (*r*) obtained between these two methods was 0,99 (see text). Green arrowhead: magnetic pulse.

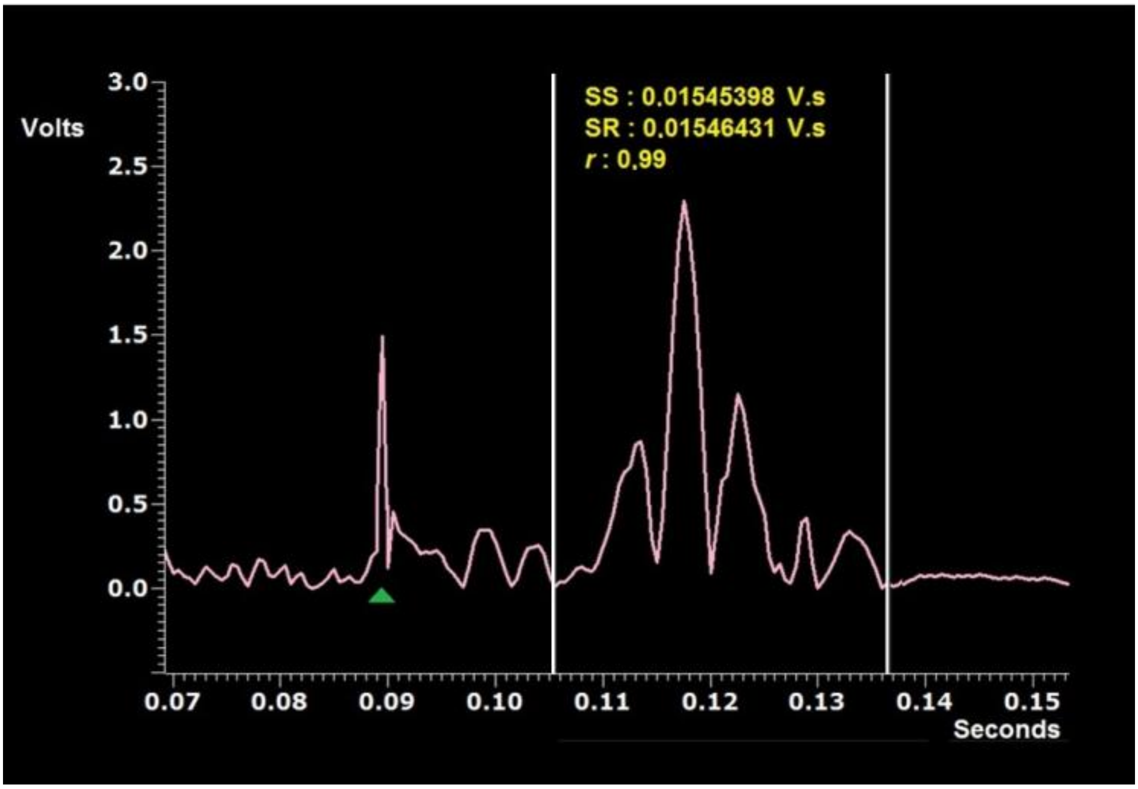

Supplementary Fig. 4. Graphical depiction of the validation made to the area of the rectified MEP in ambiguous units, or unsquared Volts times seconds, obtained a healthy human with the FDI muscle contracted. White vertical lines: region of interest. SS: values obtained automatically from Signal software®, SR: values obtained by applying the Simpson's rule. The correlation coefficient (r) obtained between these two methods was 0,99 (see text). Green arrowhead: magnetic pulse.

t	V (t)	|V(t)|	4V (t_{2n+1})	2V (t_{2n})
0.09000000	0.123047			
0.09050000	0.450439			
0.09100000	0.342285			
0.09150000	0.309570			
0.09200000	0.290039			
0.09250000	0.259766			
0.09300000	0.201416			
0.09350000	0.215820			
0.09400000	0.212402			
0.09450000	0.225586			
0.09500000	0.198486			
0.09550000	0.126221			
0.09600000	0.097412			
0.09650000	0.052979			
0.09700000	0.003662			
0.09750000	0.120605			
0.09800000	0.266602			
0.09850000	0.343994			
0.09900000	0.342041			
0.09950000	0.344971			
0.10000000	0.277100			
0.10050000	0.181641			
0.10100000	0.082275			
0.10150000	0.012451			
0.10200000	0.059326			
0.10250000	0.163330			
0.10300000	0.237549			
0.10350000	0.240479			
0.10400000	0.251953			
0.10450000	0.202148			
0.10500000	0.085205			

0.10550000	0.003662	0,003662		
0.10600000	0.037109	0,037109	0,148436	
0.10650000	0.035889	0,035889		0,071778
0.10700000	0.069824	0,069824	0,279296	
0.10750000	0.111084	0,111084		0,222168
0.10800000	0.125977	0,125977	0,503908	
0.10850000	0.10498	0,10498		0,20996
0.10900000	0.104004	0,104004	0,416016	
0.10950000	0.152344	0,152344		0,304688
0.11000000	0.250732	0,250732	1,002928	
0.11050000	0.34082	0,34082		0,68164
0.11100000	0.46167	0,46167	1,84668	
0.11150000	0.62207	0,62207		1,24414
0.11200000	0.689453	0,689453	2,757812	
0.11250000	0.717529	0,717529		1,435058
0.11300000	0.852783	0,852783	3,411132	
0.11350000	0.867676	0,867676		1,735352
0.11400000	0.698242	0,698242	2,792968	
0.11450000	0.291016	0,291016		0,582032
0.11500000	0.150635	0,150635	0,60254	
0.11550000	0.414795	0,414795		0,82959
0.11600000	0.979736	0,979736	3,918944	
0.11650000	1.580.322	1,580322		3,160644
0.11700000	2.055.664	2,055664	8,222656	
0.11750000	2.302.734	2,302734		4,605468
0.11800000	2.102.539	2,102539	8,410156	
0.11850000	1.790.039	1,790039		3,580078
0.11900000	1.260.254	1,260254	5,041016	
0.11950000	0.622559	0,622559		1,245118
0.12000000	0.087158	0,087158	0,348632	
0.12050000	0.348389	0,348389		0,696778
0.12100000	0.634521	0,634521	2,538084	
0.12150000	0.666992	0,666992		1,333984
0.12200000	0.917969	0,917969	3,671876	
0.12250000	1.148.926	1,148926		2,297852
0.12300000	1.057.861	1,057861	4,231444	
0.12350000	0.852783	0,852783		1,705566
0.12400000	0.611328	0,611328	2,445312	
0.12450000	0.529053	0,529053		1,058106
0.12500000	0.427246	0,427246	1,708984	
0.12550000	0.183105	0,183105		0,36621
0.12600000	0.094238	0,094238	0,376952	
0.12650000	0.145264	0,145264		0,290528
0.12700000	0.050537	0,050537	0,202148	
0.12750000	0.032471	0,032471		0,064942
0.12800000	0.127686	0,127686	0,510744	
0.12850000	0.388916	0,388916		0,777832
0.12900000	0.412354	0,412354	1,649416	
0.12950000	0.159424	0,159424		0,318848
0.13000000	0.000244	0,000244	0,000976	
0.13050000	0.040771	0,040771		0,081542
0.13100000	0.094971	0,094971	0,379884	
0.13150000	0.161865	0,161865		0,32373
0.13200000	0.239014	0,239014	0,956056	
0.13250000	0.310303	0,310303		0,620606
0.13300000	0.338379	0,338379	1,353516	
0.13350000	0.307617	0,307617		0,615234
0.13400000	0.286133	0,286133	1,144532	
0.13450000	0.241455	0,241455		0,48291
0.13500000	0.169922	0,169922	0,679688	
0.13550000	0.09375	0,09375		0,1875
0.13600000	0.001465	0,001465	0,00586	
0.13650000	0.03125	0,03125		
0.13700000	0.010254		61,558592	31,129882
0.13750000	0.020508			
0.13800000	0.052002			

0.13850000	0.064941
0.13900000	0.066650
0.13950000	0.060059
0.14000000	0.083008
0.14050000	0.089844
0.14100000	0.086426
0.14150000	0.078613
0.14200000	0.076904
0.14250000	0.096436
0.14300000	0.099609
0.14350000	0.089844
0.14400000	0.082764
0.14450000	0.074219
0.14500000	0.070068
0.14550000	0.052490
0.14600000	0.039063
0.14650000	0.010498
0.14700000	0.002686
0.14750000	0.005127
0.14800000	0.023193
0.14850000	0.040527
0.14900000	0.055664
0.14950000	0.048828
0.15000000	0.055908

Simpson'rule: 0,015453898

$$\int_a^b V(t)\,dt = \frac{\Delta t}{3}\left[V(t_0)+4V(t_1)+2V(t_2)+4V(t_3)+2V(t_4)+\cdots+2V(t_{n-2})+4V(t_{n-1})+V(t_n)\right]$$

Signal® Software

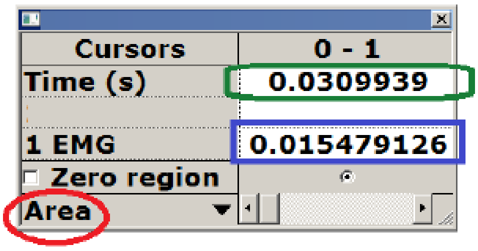

t	V(t)	|V(t)|	4V (t_{2n+1})	2V (t_{2n})

Supplementary Fig. 5. Example of the numerical method followed to validate the computation of the area of the rectified neural signal using ambiguous units, or unsquared Volts times seconds. Numbers highlighted in yellow color (left column) correspond to the region of interest selected to calculate the area of the rectified MEP. Numbers highlighted in green, blue and pink correspond to the values obtained to calculate the area by using the Simpon´s rule, which is depicted in dark blue color. A screenshot of the Signal® software is also inserted that included the most salient information automatically gave by the software used to know the area of the rectified MEP. Time: green line, voltage: blue line, area measured in ambiguous units, or Volts times seconds: red line. Note that the values obtained in ambiguous units, or Volts times seconds, of the area of the rectified MEP obtained by the Signal® software (numbers in purple color) and those obtained by the Simpsom´s rule (numbers in red color) were similar, and strongly correlated (r: 0,99, see text). In brown color: t: time in seconds, V: voltage in Volts, ||: absolute value.

	MALE		FEMALE		ALL	
	AV	Log 10	AV	Log 10	AV	Log 10
Bioenergy rMT	511,4<br>57,7	2,499721745<br>1,761284799	2326,7<br>316,0	3,3667473<br>2,499721745	1419,1<br>113,9	3,152018302<br>2,056821873
Bioenergy rMT+10	2110,9<br>127,1	2,664693709<br>2,104220104	6902,5<br>462,0	3,839009899<br>2,664693709	4506,7<br>175,0	3,653864844<br>2,243146591
Bioenergy rMT+20	8965,7<br>509,8	2,960386303<br>2,707416692	17480,4<br>912,8	4,242553724<br>2,960386303	13223,1<br>376,5	4,121334192<br>2,575795221
Bioenergy aMT	4490,6<br>291,8	2,976418562<br>2,46510351	10337,9<br>947,1	4,014433241<br>2,976418562	7414,2<br>352,0	3,870069914<br>2,546636055
Bioenergy aMT+10	64514,8<br>1660,6	3,139704817<br>3,220271548	57239,5<br>1379,4	4,757696457<br>3,139704817	60877,2<br>758,9	4,784454905<br>2,880225874
Bioenergy aMT+20	79507,7<br>1359,1	3,137393251<br>3,133269663	76698,7<br>1372,1	4,884788365<br>3,137393251	78103,2<br>676,1	4,892669158<br>2,830056915
Biowork MT	3979,1<br>302,6	3,010082446<br>2,481003314	8011,1<br>1023,4	3,903696747<br>3,010082446	5995,1<br>375,7	3,777802123<br>2,574864431
Biowork MT+10	62403,9<br>1646,3	3,162250515<br>3,216529452	50337,0<br>1452,9	4,701887562<br>3,162250515	56370,4<br>778,4	4,751051646<br>2,891241703
Biowork MT+20	70541,9<br>1165,1	3,231574611<br>3,066363708	59218,2<br>1704,4	4,772455708<br>3,231574611	64880,1<br>731,9	4,812111723<br>2,86445408

Supplementary Table: Data obtained in absolute values (AV), and in decadic logarithm (Log 10) transformed.

SUPPLEMENTARY DISCUSSION

Brain motor cortex volume differences between genders are known to exist; however, such differences do not correlate with TMS measures [1]; hence, they do not explain our bioenergy results. Corticospinal integrity and connectivity begins to change as early as 20 years-old in humans [2] making this aspect too broad to explain our results as well. Loss of alpha motoneurons is evident until the seventh decade making this mechanism also very unlikely to be a key cofactor involved in bioenergy findings. Similar situation happens with sarcopenia, which becomes evident at the age of 70 [3]. Likewise, dynapenia, the age-associated loss of strength due to the lack of intrinsic force-generating properties of skeletal muscle not caused by neurologic or muscular diseases, is more evident in healthy people older than 80 years-old [4]. Female hormones known to modulate neural transmission might also be invoked to explain the increased bioenergy found in women [5,6]. However, a recent study found decreased MEP amplitudes in females that were recorded from the abductor pollicis muscle; such findings were explained by disturbed inhibitory mechanisms that happen within the female brain motor cortex during anovulation [5]. Germane to this study, the area of the rectified MEP of the FDI to TMS during the menstrual cycle was recently reported; however, the MEP area was assessed by the ambiguous method of millivolts times seconds hindering it further comparisons with our data [7]. The afore summarized findings indicate that gender differences in our study were not a matter of body size, aging or hormone females. Thus, alternative mechanisms have to be sought.

Interestingly, downregulation of the firing of corticospinal motoneurons could be less in males by selective modulation of testosterone on GABAA-receptor [8]. Testosterone also decreases motoneuronal firing rates, which are lower in men [9]. Men also have less slow fibers in muscles than women, particularly type I fibers [10], which are most of the fibers forming the FDI muscle. Type I fibers are controlled in turn by the more smaller motor units, which are more prevalent in younger people [11]. Such fiber distribution does not disturb strength in old people, regardless gender [3], according to the onion-skin motor unit control scheme that rules FDI biomechanics [12]. In this scheme, low-threshold motor units produce more force at lower input excitation levels [12], as it was seen in the female group studied here. Should the input

increase the higher threshold motor units would be recruited. The interactions and mechanisms discussed would favor female FDI bioenergy and likely efficiency [2,13] by peripheral rather than corticospinal D and I-waves recruitment mechanisms invoked elsewhere in MEP facilitation of monkeys and man [2,14]. Indeed, corticospinal MEP recruitment induced by TMS at frequency and intensities similar to those applied in this study showed similar trends between genders; however, electrical stimulation of the ulnar nerve elicited a significant larger compound muscle action potential from FDI muscle in females than in males [2]. These findings may reflect the daily preferential use and exercise made by women of the dominant FDI muscle during thousands of years, resulting this behavioral adaptation in more effective force and biowork production at lower firing rates [6].

Although this study was not planned to disentangle the physiology of MEP but to mathematically unveil the bioenergy within a rectified neuromagnetic signal, the evidence extracted from this work indicates that large muscles such as those found in some men not necessarily contain more bioenergy than short muscles classically found in some women, regardless age [2,15]. More importantly, our novel assessment of the energy contained within the MEP complements behavioral studies that put forward that women are stronger and, sometimes, more efficient than men, at least in some neural connections [16].

Notably, bioenergy from rectified MEPs allowed assessing mechanical muscle biowork as well. This is another input this study gives to biomechanics studied by TMS. Since MEP assessments have been done elsewhere in ambiguous units (e.g., arbitrary units, uV, uV.s) [17,18] comparisons between those questionable methods and our computed biowork could be misleading. However, some conclusions could be drawn from heart muscle mechanics since the biowork extracted from the FDI muscle follows neurophysics principles employed by heart muscles while pumping blood [19] First, mechanical heart work is the delta (Δ) of energy found between the “active” state of the muscle, or systole, and the “relaxed” state of the same muscle, or diastole [19,20]. This mechanical work can be measured regardless electrocardiographic action potentials fractality and variability [21]. Although these latter aspects (e.g., MEP variability) are not the focus of this research and will not be discussed in detail here; suffice it to say

that such aspects do not influence MEP recordings elicited from the dominant FDI muscle [2] making our findings unique. Second, heart muscle work does not rely on classical impedance or resistance canonically defined for calculating electronic circuits but on refined proteostasis mechanisms [22]. Analog mechanisms would explain the biowork made by the FDI muscle, which converts endergonic energy into mechanical work adopting nanomolecular proteins and resources similar to those used by the heart [22,23].

References

[1] R.C. Gur, F.M. Gunning-Dixon, B.I. Turetsky, W.B. Bilker and R.E. Gur, “Brain region and sex differences in age association with brain volume: a quantitative MRI study of healthy young adults.” *American Journal of Geriatric Psychiatry* vol. 10, pp. 72 – 80, 2002.

[2] J.B. Pitcher, K.M. Ogston and T.S. Miles, “Age and sex differences in human motor cortex input-output characteristics.” *Journal of Physiology* vol. 546, no. 2, pp. 605 - 613, 2003

[3] J. Lexell, K. Henriksson-Larsén, B. Winblad and M. Sjöström, “Distribution of different fiber types in human skeletal muscles: effects of aging studied in whole muscle cross sections.” *Muscle and Nerve* vol. 6, no. 8, pp. 588 – 595, 1983.

[4] T.M. Manini and B.C. Clark, “Results from a Web-based survey to identify dynapenia screening tools and risk factors.” *Journal of Cachexia Sarcopenia Muscle* vol. 7, no. 4, pp. 499 – 500, 2016.

[5] A.V. Peterchev, T.A. Wagner, P.C. Miranda, M.A. Nitsche, W. Paulus, S.H. Lisanby et al, “Fundamentals of transcranial electric and magnetic stimulation dose: definition, selection, and reporting practices.” *Brain Stimulation,* vol. 5, no. 4, pp. 435 – 453, 2012.

[6] K. Hattemer, S. Knake, J. Reis, J. Rochon, W.H. Oertel, F. Rosenow et al. “Excitability of the motor cortex during ovulatory and anovulatory cycles: a transcranial magnetic stimulation study.” *Clinical Endocrinology,* vol. 66, no. 3, pp. 387 – 393, 2007.

[7] M. Zoghi, B. Vaseghi, A. Bastani, S. Jaberzadeh and M.P. Galea, “The effects of sex hormonal fluctuations during menstrual cycle on cortical excitability and manual dexterity.” *PLoS ONE* vol. 10, no. 8, pp. e0136081, 2015.

[8] M. Wang, “Neurosteroids and GABA-A receptor function.” *Frontries in Endocrinology,* vol. 2, pp. 44, 2011.

[9] J.J. Hulmi, J.P. Ahtiainen, H. Selänne, J.S. Volek, K. Häkkinen, V. Kovanen et al. “Androgen receptors and testosterone in men--effects of protein ingestion, resistance

exercise and fiber type." *Journal of Steroid Biochemical Molecular Biolology,* vol. 110, no. 1-2, pp. 130 – 137, 2008

[10] M. A. Johnson, G. Sideri, D. Weightman and D. Appleton, "A comparison of fibre size, fibre type constitution and spatial fibre type distribution in normal human muscle and in muscle from cases of spinal muscular atrophy and from other neuromuscular disorders." *Journal of the Neurological Sciences* vol. 20, no. 4, pp. 345 – 361, 1973.

[11] T.J. Doherty and W.F. Brown, "The estimated numbers and relative sizes of thenar motor units as selected by multiple point stimulation in young and older adults." *Muscle and Nerve,* vol. 16, no. 4, pp. 355 – 366, 1993.

[12] J.O. Ortega, S.L. Lindstedt, F.E. Nelson, S.A. Jubrias, M.J. Kushmerick and K.E. Conley, "Muscle force, work and cost: a novel technique to revisit the Fenn effect." *Journal of Experimental Biology*, vol. 218, no. 13, pp. 2075 – 2082, 2015.

[13] C.J. Barclay and C.L. Weber, "Slow skeletal muscles of the mouse have greater initial efficiency than fast muscles but the same net efficiency." *Journal of Physiology,* vol. 559, no. 2, pp. 519 – 533, 2004.

[14] E. Palmer and P. Ashby, "Corticospinal projections to upper limb motoneurones in humans." *Journal of Physiology,* vol. 448, pp. 397 – 412, 1992

[15] R.A. Conwit, D. Stashuk, B. Tracy, M. McHugh, W.F. Brown and E.J. Metter, "The relationship of motor unit size, firing rate and force." *Clinical Neurophysiology,* vol. 110, no. 7, 1270 - 1275, 1999

[16] M. Filippi, P. Valsasina, P. Misci, A. Falini, G. Comi and M.A. Rocca, "The organization of intrinsic brain activity differs between genders: a resting-state fMRI study in a large cohort of young healthy subjects." *Human Brain Mapping*, vol. 34, no. 6, pp. 1330 – 1343, 2013

[17] A. Rotenberg, P.A. Muller, A.M. Vahabzadeh-Hagh, X. Navarro, R. López-Vales, A. Pascual-Leone and F. Jensen, "Lateralization of forelimb motor evoked potentials by transcranial magnetic stimulation in rats." *Clinical Neurophysiology,* vol. 121, no. 1, pp. 104 – 108, 2010.

[18] D. Winter, "Units, terms and standards in the reporting of EMG Research". Report by the Ad Hoc Committee of the International Society of Electrophysiological Kinesiology. Montreal: Rehabilitation Institute of Montreal, Canada, pp. 1–16, 1980.

[19] N. Westerhof, N. Stergiopulos and M.I.M Noble, "Snapshots of Hemodynamics: An Aid for Clinical Research and Graduate Education." Springer, Berlin, Germany, 2010.

[20] R.A. Serway and J.W. Jewett, "Physics for Scientists and Engineers with Modern Physics." Wadsworth, Boston, MA, 2013.

[21] D.C. Lin and A. Sharif, *"*Common multifractality in the heart rate variability and brain activity of healthy humans.*" Chaos*, vol. 20, pp. 023121, 2010

[22] R. Yang, Y. Qin, C.Li, G. Zhu and Z.L. Wang, "Converting biomechanical energy into electricity by a muscle-movement-driven nanogenerator." *Nano Letters,* vol. 9, no. 3, pp. 1201 – 1205, 2009.

[23] F.E. Leon-Sarmiento, J.S. Leon-Ariza, D.G. Prada, D.S. Leon-Ariza, "Chemosensory disturbances-associated nanocholinergic dysfunction: The case of, not only, myasthenia gravis." *Journal of the Neurological Sciences*, vol. 356, no. 1-2, pp. 5–6, 2015
